# Spinal Cord Stimulation as Treatment for Cancer and Chemotherapy-Induced Pain

**DOI:** 10.3389/fpain.2021.699993

**Published:** 2021-08-24

**Authors:** Breanna L. Sheldon, Jonathan Bao, Olga Khazen, Julie G. Pilitsis

**Affiliations:** ^1^Department of Neuroscience and Experimental Therapeutics, Albany Medical College, Albany, NY, United States; ^2^Department of Neurosurgery, Albany Medical Center, Albany, NY, United States

**Keywords:** spinal cord stimulation, chronic pain, cancer pain, neuropathic pain, chemotherapy-induced pain, neuromodulation

## Abstract

Neuropathic pain is a rampant disease exacting a significant toll on patients, providers, and health care systems around the globe. Neuromodulation has been successfully employed to treat many indications including failed back surgery syndrome (FBSS), complex regional pain syndrome (CRPS), phantom limb pain (PLP), radiculopathies, and intractable pelvic pain, among many others. Recent studies have also demonstrated efficacy for cancer-related pain and chemotherapy induced neuropathy with these techniques. Spinal cord stimulation (SCS) is the most commonly employed technique and involves implantation of percutaneous or paddle leads targeting the dorsal columns of the spinal cord with the goal of disrupting the pain signals traveling to the brain. Tonic, high frequency, and burst waveforms have all been shown to reduce pain and disability in chronic pain patients. Closed-loop SCS systems that automatically adjust stimulation parameters based on feedback (such as evoked compound action potentials) are becoming increasingly used to help ease the burden placed on patients to adjust their programming to their pain and position. Additionally, dorsal root ganglion stimulation (DRGS) is a newer technique that allows for dermatomal coverage especially in patients with pain in up to two dermatomes. Regardless of the technique chosen, neuromodulation has been shown to be cost-effective and efficacious and should be given full consideration in patients with chronic pain conditions.

## Introduction

Chronic pain is a rampant disease affecting large swaths of global populations. The 2016 Global burden of disease study found that pain-related conditions, especially low-back and neck pain, are the leading cause of disability worldwide (1). Defined as intractable pain lasting >3 months, chronic pain is complex and difficult to treat. Neuropathic pain, a subtype of chronic pain, is related somatosensory nerve dysfunction and does not generally respond to opioid medications (2). Spinal cord stimulation (SCS) and dorsal root ganglion stimulation (DRGS) are two neuromodulatory techniques that have been shown to effectively treat neuropathic pain.

SCS is a form of neuromodulation that targets the spinal cord with electrical impulses to treat various pain conditions. It was first clinically utilized in 1967 by Shealy et al. to treat chronic pain induced by metastatic lung cancer (3). Since then, its applications include failed back surgery syndrome (FBSS), angina, critical limb ischemia, neuropathic pain and complex regional pain syndrome (CRPS) (4–8). The mechanism of action of SCS was based on the gate control theory postulated by Melzack and Wall in 1965, which proposed that stimulation of Aβ fibers could attenuate or suppress pain signals traveling through Aδ and C fibers (9). However, new waveform stimulation techniques such as high-frequency stimulation (HFS) and burst stimulation have challenged this understanding, and the precise mechanism is much more nuanced (10–13). SCS usage has grown despite this gap in knowledge (4–6, 14, 15), likely in part because it is cost-effective compared to conventional medical management and primary spine surgery (16–20).

The success in treating other chronic pain disorders has led to SCS's use in treating cancer and chemotherapy-induced pain. Cancer-related pain affects more than 70% of patients and up to 40% of these patients report experiencing neuropathic pain (21). In multiple reports and retrospective reviews, SCS has been demonstrated to effectively treat cancer-related pain (22–27). Preliminary evidence from small case studies note significant improvements in pain relief from neuromodulation in patients with metastatic bone disease (28, 29). It has been also employed to treat chemotherapy-induced neuropathy (CIN) (22, 23). Cata et al. conducted a case study of two patients with CIN refractory to medication, who completed a successful trial of SCS and proceeded to permanent implant. Both patients showed improvement in pain scores, gait, flexibility, touch, sharpness detection, and reduction in medication, and improvements in gait and flexibility (23). Abd-Elsayed et al. similarly reports a case study in which a patient with breast cancer and CIN had excellent pain relief, improvement in daily functioning and sleep, and reduction of pain medication following SCS (22). CIN occurs in up to 40% of patients treated with agents known to have the potential for neurotoxicity and has been shown to significantly increase cancer survivor morbidity and healthcare expenditures (30). Given that pain and motor dysfunction are the most common reasons for discontinuation of chemotherapy prior to course completion—-particularly with the frequently used platinum-containing compounds, vinca alkaloids, and taxols—-SCS efficacy may be particularly significant (Table 1) (31–33). Though these initial results have been promising, large-scale RCTs have not yet been performed for this application. In this review paper, we give a general overview of SCS use, including stimulation types and novel closed-loop systems, as well as DRGS for treatment of neuropathic pain.

**Table 1:** Common chemotherapy agents with neuro-related side effects.

**Chemotherapy agent**	**Examples**	**Common uses**	**Neuro-related side effects**
Platinum-containing compounds	Cisplatin Carboplatin Oxaliplatin	Solid tumor malignancies (non-small cell lung cancer, testicular cancer, ovarian cancer, bladder cancer)	Peripheral neuropathy
Vinca alkaloids	Vincristine Vinblastine	Both hematologic (leukemias, lymphomas) and solid malignancies (pediatric tumors, breast cancer, germ cell cancer)	Neurotoxicity, including peripheral sensory neuropathy Autonomic dysfunction
Taxols	Paclitaxel Docetaxel Cabazitaxel	Breast cancer, ovarian cancer, AIDS-related Kaposi's sarcoma	Neurotoxicity, including peripheral sensory neuropathy

## Spinal Cord Stimulation: Patient Selection

**Figure 1:**
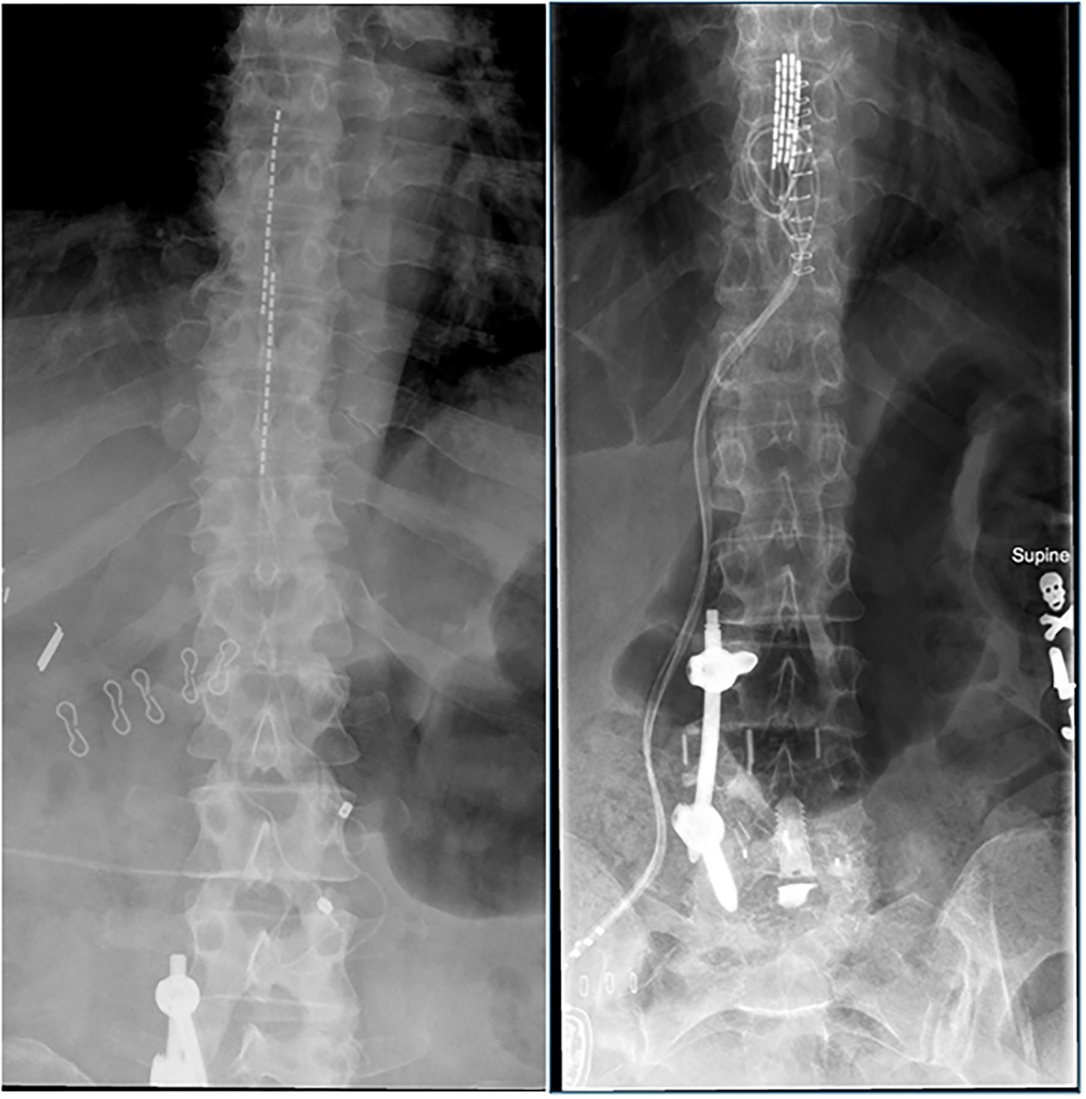
Examples of percutaneous SCS leads **(left)** and paddle SCS leads **(right)**.

When a patient has failed conservative measures and wishes to pursue SCS as a means of treatment, the first step is a 5–7 day trial with temporary leads. If >50% pain relief is achieved with the trial, the patient is considered for permanent implantation (34). In general, SCS devices consist of two parts: electrodes which deliver the electric impulses and an implantable pulse generator (IPG) that serves as the power source and pacemaker. Leads for these devices can either be directly implanted via a small laminectomy to place a paddle, or percutaneously (Figure 1). IPGs may either be rechargeable or non-rechargeable. The physical footprint of the IPG is larger for non-rechargeables, but they have less patient burden and are less expensive. Rechargeable IPGs last two to three times as long as non-rechargeables. Charging IPGs varies based on the amount of energy consumed but in general patients should charge their devices at least once a week for about 1 h by placing a charger over skin at the battery site. This process can become burdensome and sometimes painful. There are two options for intraoperative feedback required during surgery. Intraoperative mapping establishes stimulation frequencies, amplitude, width, and location and requires patient feedback during the operation. Alternatively, intraoperative electromyography can be used on patients under general anesthesia to predict energy requirements and device placement (35). Either immediately or shortly after the procedure is complete, patients will meet with a company-specific device representative to program their devices for everyday use.

### Patient Selection

When preparing a patient for SCS, it is important that realistic goals are cooperatively set by provider and patient. Patients are considered “responders” if 50% or greater pain relief is achieved. A discussion carefully delineating this expectation is essential to success, and patients should not expect to be pain free after the surgery. It is also important to weigh comorbidities when determining if a patient is a candidate for the surgery. Patients with conditions causing immunosuppression or impaired wound healing require careful consideration, though successful implantation is possible (29). Studies have shown permanent pacemakers and implantable cardioverter defibrillators can be safely used in conjunction with SCS systems, though close follow-up and coordination with the patient's cardiologist is recommended (36). Comorbid psychiatric conditions such as major depressive disorder and anxiety disorders, which are particularly common within the chronic pain population, should be similarly optimized prior to surgery to maximize pain relief (37–39). Finally, tobacco use diminishes the responder rates of SCS, and providers should consider discussing smoking cessation with patients prior to impantation (40).

## Types of SCS Stimulation

### Tonic Stimulation

Tonic stimulation is the conventional waveform used in most initial clinical studies of SCS. This form of SCS employs a consistent, low frequency stimulation generally between 40 and 60 Hz to generate burning, tingling sensations. These “paresthesias” ideally should overlap the areas of pain to mask and replace the perception of pain at that location (41). The landmark PROCESS clinical trials conducted by Kumar et al. found tonic SCS provided superior pain relief and quality of life as compared to traditional medical management when treating FBSS (14). Since then, multiple RCTs have shown that tonic stimulation improved chronic pain related CRPS and DPN as well (7, 42–44). In treating CRPS, Kemler et al. reported greater reductions in the visual analog scale (VAS) for pain when treated with tonic SCS as compared to physical therapy (43, 44). At the 5-year endpoint of the study, the change in VAS scores remained higher in the SCS group, but the was no longer statistically significant. However, patients who underwent SCS still reported satisfaction with the treatment and stated that they would go through this treatment again for the same results. Tonic SCS also demonstrated greater pain relief as compared to traditional medical management of diabetic neuropathy with 60% of SCS patients meeting success criteria at 6 months as compared to only 5–7% in the medical management group (7, 42).

While the above advancements have yielded relief for many patients, tonic SCS' attainable benefit is variable. For example, this waveform works well for treatment of limb pain but has in the past faced challenges in treating chronic back pain, where adequate paresthesia coverage may be difficult to achieve (45–48). Paresthesia is also poorly tolerated in some populations and may not provide pain relief (34). Finally, a number of review papers imply the true mechanisms underlying this waveform are much more complex at both the cortical and segmental levels than previously thought (49, 50). Tonic SCS may be further developed to improve patient outcomes as more exact mechanisms are elucidated.

### High-Frequency Stimulation

HFS is a form of SCS that utilizes higher frequencies and lower amplitudes as compared to tonic SCS and was part of the second phase of SCS advancements searching for paresthesia-free SCS. Notably, HFS does not produce paresthesia, making it a treatment option for patients who do not tolerate paresthesia associated with tonic stimulation (51, 52). The majority of studies have investigated HFS as a treatment for FBSS and more recently DPN (12, 13, 53–55). A large RCT was conducted by Kapural et al. comparing HFS at 10,000 kHz (HF10) to tonic SCS found HF10 had a higher response rate at 80%, improvements in opioid consumption rates, disability, and satisfaction at 12 months, and no differences in complication rates (12). Importantly, HF10 also maintained better pain relief in both the back and leg at a 24-month follow-up (13). Despite these notable findings, HFS is not well-understood. Proposed mechanisms from various preclinical studies include blockage of axonal conduction, alterations of glial-neuronal interactions, and disruptions of axonal activity (56). Further research is warranted to fully understand its clinical efficacy and expand its usage.

### Burst Stimulation

Burst stimulation involves 500 Hz of stimulation delivered in bursts of 5 at a frequency of 40 Hz which is hypothesized to mimic neuronal firing. Burst SCS minimizes paresthesia similarly to HFS and thus can be utilized in patients who do not tolerate this (10, 11). Multiple studies support that burst SCS produces similar, if not superior, pain relief as compared to tonic SCS (10, 11, 57). In the SUNBURST RCT, burst SCS produced superior results to tonic SCS for the treatment of FBSS or neuropathic radicular pain, such that an intention-to-treat analysis found burst stimulation to be non-inferior (*p* < 0.001) and superior (*p* = 0.017) to tonic stimulation (58). Furthermore, patients demonstrated a clear preference for the burst SCS (70.8%) which was due in part to improved pain relief reported by some patients; this preference was maintained at the one-year follow-up (58). Smaller studies with crossover designs have demonstrated that burst stimulation produces greater improvement in pain scores as compared to tonic stimulation and placebo (10, 57).

Interestingly, burst SCS may activate certain pain centers contributing to its analgesic effects. De Ridder et al. demonstrated pain vigilance and awareness which the tonic stimulation and placebo decreased, possibly attributable to increased activation of the dorsal anterior cingulate cortex and right dorsolateral prefrontal cortex observed encephalogram following burst stimulation (10). While this study may have potential confounding effects with the various treatments given, it describes a potential mechanism of action. Further study by De Ridder and colleagues found that burst stimulation also increases activity in other brain regions like the pregenual anterior cingulate cortex, posterior cingulate cortex, and parahippocampus (10, 47). Activation of these combined regions may lead to modulation of descending inhibitory pathways and affective pain perception, which may account for burst SCS' analgesia as well as effects on mood (47).

### Closed-Loop SCS Systems

The intensity of a patient's stimulation varies depending on the distance between the implanted leads and the dorsal columns of the spinal cord. Anything that affects this distance can therefore affect how stimulation is felt, leading to either too much stimulation causing discomfort or too little stimulation to be efficacious (59). This includes positional changes, different activities that may increase the amount of pain felt, and body functions like coughing, sneezing, or laughing. To adjust for these variations, device representatives generally create 2–3 “programs” for patients to toggle between as needed to achieve appropriate stimulation during daily activities. However, closed-loop systems will automatically adjust the settings, allowing patients to perform daily activities without having to worry about changing their programs.

In contrast, cardiac pacemakers use feedback from the target organ to alter delivery of electrical impulses. More recent closed-loop SCS systems also can automatically adjust stimulation parameters based on feedback mechanisms with the aim of providing more consistent stimulation delivery. These could theoretically abolish the need for multiple programs and decrease the amount of patient participation required for success with SCS, which in turn will foster improved long-term compliance and increase achievable benefits postoperatively. Two feedback mechanisms currently under study for use in closed-loop SCS systems are discussed below.

### Accelerometry

As mentioned above, common daily movements like standing, sitting, walking, or lifting can affect the level of stimulation felt by the patient. Accelerometry can be used to detect changes in posture. A small study of 15 patients showed that 74% of persons using an SCS system that automatically changed stimulation parameters with positions changes reported that the paresthesias felt were “just right” (60). Following this, Schultz et al. conducted a prospective, randomized study featuring a crossover design (61). This open-label study demonstrated greater pain relief and decreased manual programming requirements when the accelerometry-based SCS system was in use (61). It also demonstrated further evidence that patients were more satisfied with less intervention required on her parts. Further, these accelerometry-based systems may be a step in the right direction at achieving the “goldilocks” level of stimulation for patients.

### Evoked Compound Action Potentials

More recently, ECAPs have been used to close the loop. ECAPs represent the cumulative action potential generated in response to stimulation and can be used to quantify a nerve's response to SCS (62). Relying on ECAPs for feedback allows for responses to fluctuate based on the distance between SCS leads and the spinal cord, such as those caused by respiration and cardiac activity. The recent Avalon RCT established the efficacy of an ECAP-based closed-loop system, demonstrating an impressive responder rate of 89.5% across the 38 subjects completing the full 24-month study and no unanticipated complications or safety concerns (63). However, the single-arm treatment design limits the conclusion providers can draw as to whether the device itself is superior. The Evoke study did feature an open-loop cohort as a control, and significantly more patients implanted with the ECAP-based SCS system were responders at the 3-month and 12-month timepoints, respectively (64). Both studies demonstrated similar safety profiles with the ECAP system as compared to open-loop SCS (63, 64). While further validation is needed, these novel closed-loop systems may offer solutions to inconsistent stimulation in SCS patients.

## Dorsal Root Ganglion Stimulation

DRGS is a similar method of neuromodulation to SCS with one important difference: the target of the leads. Instead of placing leads to stimulate the dorsal columns (50), percutaneous leads target the dorsal root ganglion (DRG) where sensory neuronal bodies are housed. This theoretically allows for precise targeting of specific pathological neurons that may be firing aberrantly and causing pain. This mode of neuromodulation has been particularly effective for neuropathic pain conditions including postsurgical pain, CRPS, and phantom limb pain (PLP) (65–67). Case reports and retrospective studies have also demonstrated efficacy treating refractory pelvic pain and postherniorrhaphy pain (68–70). The ACCURATE trial conducted by Deer et al. is a prospective RCT that demonstrated evidence that DRGS achieved higher responder rates as compared to SCS for CRPS and lower limb causalgias (65). This study also supported previous findings of more precise paresthesia coverage achievable with DRGS as compared to SCS, particularly in the ever-challenging regions to target like the foot and lower back (65, 71, 72). However, pain relief was also achieved without paresthesia coverage in the ACCURATE trial, and subsequent retrospective studies recommend that DRGS should aim just below the sensory threshold (73, 74). Additionally, the absence of a significant epidural space limits variation with position (65, 72). The most commonly cited concerns with DRGS are related to the operator, as it is a relatively new procedure that is more technically demanding compared to SCS (66). Other concerns lie in the ability to easily remove the leads. A review of complications following DRG surgery cited lead removal as a common complication (75). Additionally, a case study on 4 patients undergoing DRG for various indications noted lead fracture in all four cases, where 3 of the 4 have undergone previous surgeries (76). Regardless, DRGS is an area of active study.

Research examining DRGS for a variety of pain indications, including cancer-related pain is underway. Studies examining vincristine-induced neuropathy in animal models have demonstrated increased pain tolerance with stimulation of the DRG with ultrasound waves (77, 78). An investigation by Koetsier et al. in animal models with DPN demonstrated that DRGS' effects are not related to GABA release from inhibitory neurons in the dorsal horn (79). This suggests that the mechanism by which DRGS alleviates neuropathic pain is distinct from SCS. While confirmatory studies in larger animal and human subjects are still in the pipeline, DRGS may therefore be a particularly fruitful treatment of cancer-related pain in the future.

## Alternative Procedures

Other surgical procedures for cancer pain involve interruptions of pain pathways by lesioning nerves, nerve roots, ganglion, etc. These procedures include spinal cordotomy, midline myelotomy, and thalamotomy. Spinal cordotomy is the most common and it eliminates pain sensations from the opposite side of the body (80). Lesion procedures provide immediate pain relief and are useful for patients suffering with malignant pain. However, there are limitations. Cordotomy is irreversible, may result in unpleasant side effects like numbness and weakness and bilateral cases may result in breathing difficulties (80). Most important is that the relief lasts between 3 and 12 months so for non-malignant pain, this may not be adequate. Risk and benefit profile for myelotomy is similar. Brain ablation results tend to last longer but which patients may benefit is often difficult to predict (80).

## Cost-Effectiveness of SCS

Chronic pain not only takes a physical and emotional toll on the patient but it also poses a great financial burden for those suffering from it. The cost of chronic pain to society is up to $USD 635 billion due to patients' excessive utilization of healthcare resources (81). In general, chronic pain patients were found to visit the emergency department more frequently than patients without pain (82–84). More so, the treatment of chronic pain is complex and often results in patients being passed from practice to practice, accumulating bills with little to no relief. Neuromodulation has become an effective alternative treatment for patients refractory to conservative medical management (85). Neuromodulation provides sustainable pain relief for many and subsequently reduces healthcare utilization and unnecessary costs. Kumar et al. demonstrated the incremental cost-effectiveness ratio for SCS ranged from CAN$ 9,293–11,216 (16). The study also showed that SCS provided positive incremental net monetary benefits compared to conventional medical management and the probability of SCS being more cost-effective ranged from 75 to 87% based on pathology (FBSS, CRPS) (16).

## Conclusion

Neuropathic pain exacts a significant toll on patients, providers, and health care systems alike. SCS represents a cost-effective and effective treatment for a variety of neuropathic pain indications. Tonic, burst, and HFS have all shown benefit in treating various chronic pain phenotypes, including cancer-related pain and chemotherapy induced neuropathy. Closed-loop SCS systems should be evaluated with cautious optimism as potential future treatments, particularly in patients who have difficulty achieving consistent therapeutic stimulation levels. Finally, the newer DRGS is emerging as an additional therapy for those wishing to more precisely target dysfunctional neurons to mitigate pain. As research continues and neuromodulatory techniques, so too will the pain relief and disability endured by chronic pain patients.

## Author Contributions

JP designed the manuscript, provided clinical expertise, and edited the manuscript. BS drafted the manuscript with help from JB and OK. All authors approved the final manuscript.

## Conflict of Interest

JP was a consultant for Boston Scientific, Nevro, Jazz Pharmaceuticals and Abbott and receives grant support from Medtronic, Boston Scientific, Abbott, Nevro, Jazz Pharmaceuticals, GE Global Research, NIH 2R01CA166379-06, and NIH U44NS115111. She was the medical advisor for Aim Medical Robotics and Karuna and has stock equity. The remaining authors declare that the research was conducted in the absence of any commercial or financial relationships that could be construed as a potential conflict of interest.

## Publisher's Note

All claims expressed in this article are solely those of the authors and do not necessarily represent those of their affiliated organizations, or those of the publisher, the editors and the reviewers. Any product that may be evaluated in this article, or claim that may be made by its manufacturer, is not guaranteed or endorsed by the publisher.
